# Nature, the Good Physician

**Published:** 1907-03

**Authors:** J. D. Macpherson

**Affiliations:** Akron, N. Y.


### Nature, The Good Physician.

By J. D. MACPHERSON, M. D., Akron, N. Y.

VOLUMES have been written by doctors, for doctors and of doctors, but tonight I am going to speak of the silent physician whose voice is never heard, who writes no theses and who tells no lies.

I shall not speak as a champion, for he needs none, but say just a few words to recall to you the presence of the greatest healer earth has ever seen. . No gaudy front or gilded sign betrays his presence on the avenue, no clanging bell announces that the doctor is in; no hurrying footsteps, no chattering hoof, tells the quiet neighbors of the urgency of the call. Long before the best trained ambulance corps can reach the scene of emergency, old doctor nature is already there, his vast host of trained assistants laying out his plan of cure.

Where is there a doctor who would so meekly permit a gruff ambulance surgeon to wheel away his patient, patiently watch the ostentatious efforts of the surgeon as with human ingenuity he professes to assist, silently listen to the babble as to the result; then, after badly misdirected efforts of the surgeon who, without even the gratification of remonstrance, would so quietly set about to bridge the error of another?

While the mighty human hands lie still in sleep, this faithful worker patches up the errors of the day. The misplaced bone is deftly calloused in, the surgeons gash must have its pyogenic wall built without a second's wait, and thus the day and night is spent in ceaseless toil to keep the tenement of clay, our human habitation, whole. Let tired nature now but seek a moment of repose, a telegram along some racked nerve reports a microbe undermining some suburban center; perhaps the tonsil is the part which demands his skill, and there soon a battle wages fierce and strong as leukocyte and pkagoeyte unite in deadly strife. The friction of the combat makes the human battle ground so chill and ill that at this juncture Dr. Pills is called in from his slumbers and, hastening to the scene of action, fires in his dose of capsules, pills or gatling hypodermics. Slowly the mighty potion stills alike the friend and foe; then bidding waking ones good night, he returns to his slumbers thanking God for hypodermics.

Doctor, did you ever place your skilful self outside the field of contest long enough to see what nature could do? Do you fully understand that your feeble efforts are simple repetitions of what nature has done a thousand times before? Did you

---

1. Presidential address delivered at the eighty-sixth annual meeting of the Medical Society of the County of Erie, January 14. 1907.

ever watch nature perform an amputation, a version, plane off a mass of granulations, tie off a tumor, drain a lung abscess, a salpingitis, or a hopeless case of appendicitis? She may finally bring safety after all hope has been abandoned; nevertheless, as the credit is yours, you also overlook a thousand other miraculous conditions which nature cures after you have considered them quite beyond the pale of human aid.

By this little digression from stereotyped lines, I wish to draw your attention closer to nature's methods. In nearly every case she mutely tells us by unerring symptoms when she needs the aid of human hands. Men, like swine, would trample jewels cheaply bought; nature only lets us mine from her storehouse facts as fast as we become educated to appreciate them. She admitted us to the sanctity of the peritoneum only when we knew enough to approach her portals with clean hands; no prayers or incense of the previous century could avail until the rays of microscopic investigation lighted up the scene.

Nature does not want to interfere with our rights. She is the most charitable physician on earth. God pity us if she were not. All she wants or asks of us is intelligent cooperation; she wants us to be on the watch for the line of demarcation, that old line with which we are all familiar in gangrene, and we should be able to locate it in other conditions. As a country physician, perhaps we have been forced too often to strain the danger line from bare necessity; but on the other hand, do not our city compeers quite as frequently err in rushing the patient to a crisis, simply because facilities are at hand?

Abdominal surgery has become such a mine of wealth, its exploitation such a fascination to the would-be surgeon, that the inexperienced hover like a cloud of vultures round the clinic rooms. If some clinical Messiah would drive them from the operating rooms to the field of general practice for a probationary period of at least five years, commercial surgery would become the exception.

We do not wish to be placed upon record as decrying surgery. We appreciate the fact that well-timed surgery is a priceless boon to humanity. Uncalled-for surgery and useless medication, however, both are a tax upon the layman, which the medical profession has no moral right to impose. Nevertheless we are proud to state that the great mass of our profession stands shoulder to shoulder for what represents good government, good ethics, and good common sense. The public is slowly but surely coming to a correct understanding as to when operations are imperative. Five years ago a countryman could hardly be dragged to the operating table for an early appendectomy; nowadays, he often requests it. This comes from education; the public is not slow to classify results, and in the smaller towns results from a professional standpoint are often obnoxiously apparent.

Preventive medicine as well as preservative surgery are both making desperate efforts to gain their proper foothold in the arena of medical science. Preventive medicine is seen in its best light from the side of sanitary science, whose gigantic strides during the past decade have placed it in the advance guard of cure. By its grand work throughout the state, sanitary science is slowly but surely educating the masses to a correct understanding as to where danger lurks, the secret hiding places of the unseen being pointed out. As the blood upon the lintel told the ancient Jew when the destroying angel was at work, so do our sanitary placards announce to every loyal citizen of the state a statute which it is for his interest to obey.

No crusade has ever been productive of so much good in so short a time as the quiet work of the sanitary officers of the state. The rapid dissemination of printed literature among the masses; the pointing out of the true source of infection; the bringing to the notice of the laity, the possibility of prevention in typhoid and tuberculosis; the mapping out of the entire care of a consumptive, even to the minutest detail, familiarizes the untrained attendant with the means of infection. Instinct alone will teach that dead men do not win battles, as surely as does the doctor know when his patient dies his fee stops. Popular notions are fast becoming relegated to the past; the slighter ailments need some rays from the camera lucida; one gets tired listening to the history of common colds and how they were contracted; a little study of nature's processes would teach a thinking mind how foolish to believe that a draft or exposure to wet or dampness will cause a sickness. Watch yourself during the next coryza you have, noting the tiny burning spot upon the upper floor of the soft palate, also the rapidity of its spread, the general inflammation invading one nostril, then the other; soon the slight rigors, then the bonesache—that's all, the microbe is in. The draft keeps on blowing just the same; having learned the true source of the trouble, up goes your window and God's fresh air fans your fevered brow, and does not come sneaking through the casement crevices or keyhole as it did of yore. This same germ to produce the varities of so called colds or influenzas has but to change its location or extend its lines, to produce all the different grades of respiratory lesions occuring under that special class.

We often try to picture to ourselves germ life as being legion in kind as well as numbers. This we do not believe to be the case. The same germ working in a different habitat may produce totally different symptoms; and thus we take it that where germ life has each been reduced to its common denominator, those inimical to human life will be found much fewer in kind than we now suppose. Nature draws no distinct lines to separate disease into sharply defined classes, but she gives us many warnings by the presence of local lesions which are too often overlooked and which long antedate the fatal issue, but point as truly toward the aftercoming result as the guide board at the country crossroad. While we do not intend to give any distinct classification of nature's signs, here are a few which might be tacked up as distinctive of the long list: early pulmonary hemorrhages, recurring pleurisy, contracted kidney, nervous dyspepsia with its concomitant mitral lesion, confusion of speech, shuffling gait, hepatic insufficiency, and last but not least. arteriosclerosis,—these all faithfully portray oncoming intrinsic lesion.

While we do not pose as an educator, we wish to reiterate the feeling of a general practitioner that it is our duty to assist nature, not to place ourselves too distinctly in the foreground, but to anticipate nature at every point possible when she needs assistance. She has taught us lessons in promptitude by the manner in which she goes to work to expel the invader; now while eagerly watching for her signs of distress let us not overlook her warning placard, "Hands off." Nature and humanity should work hand in hand, young men and old men laboring side by side; the aggressiveness of youth toned down by the conservatism of age,—age supplying counsel and wisdom, youth, energy, impetuosity and action. The results of this combination has already made the research work of the nineteenth century gleam like a star of the first magnitude against the dark background of the past six hundred years.

